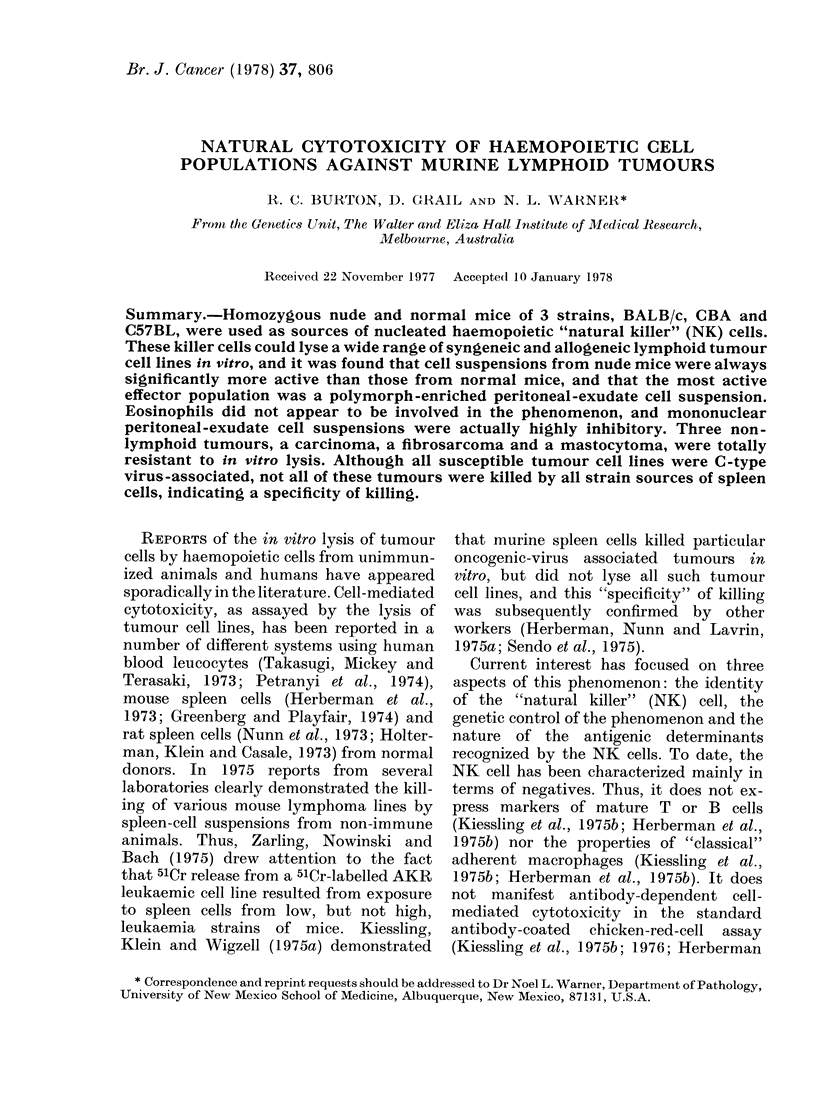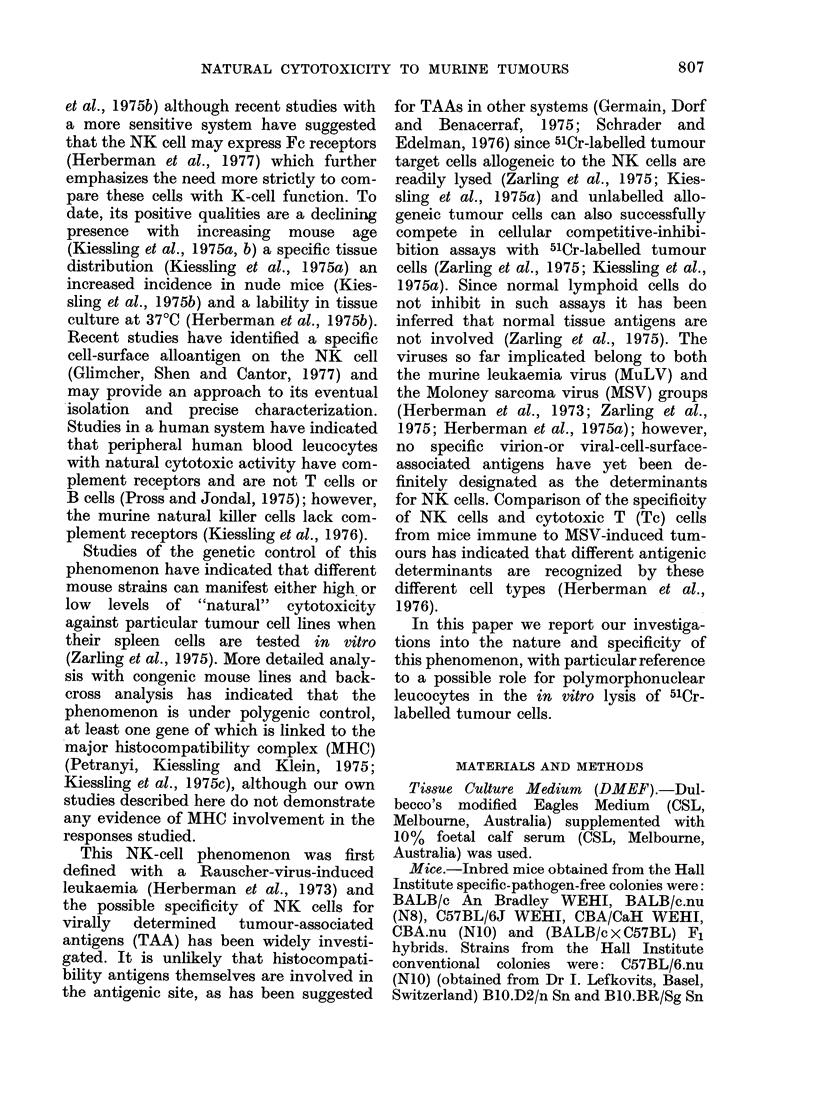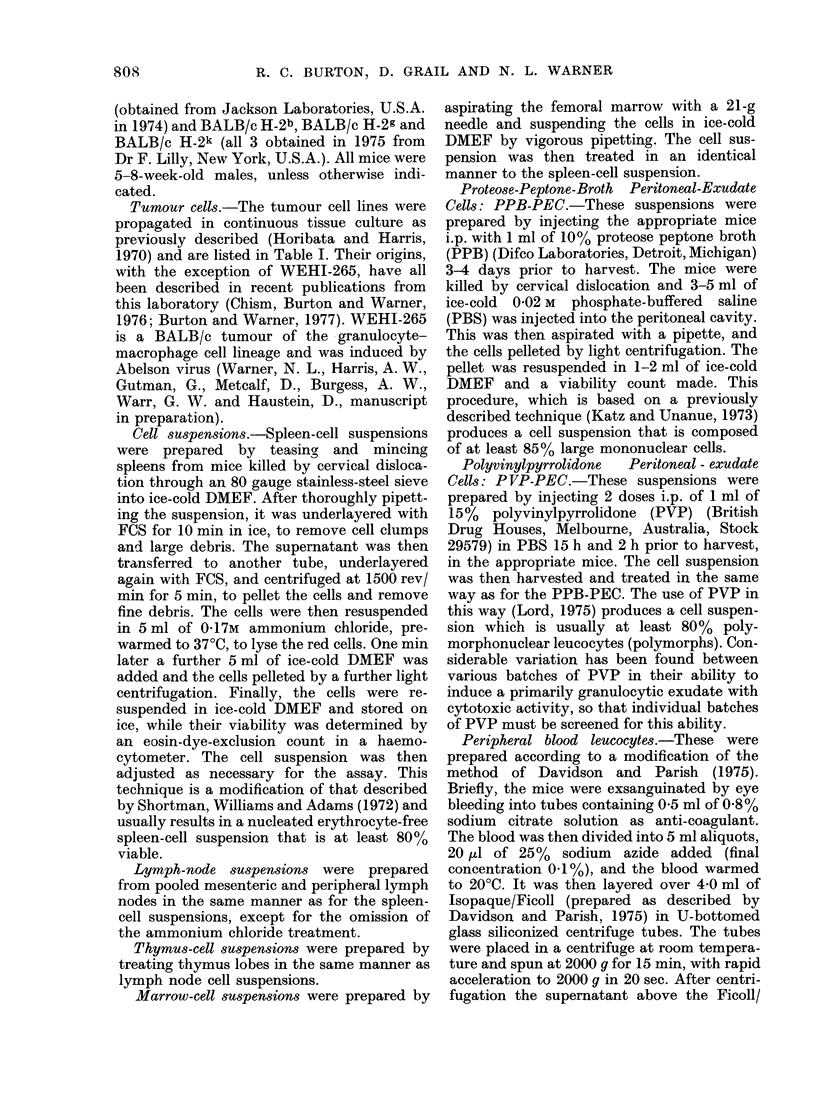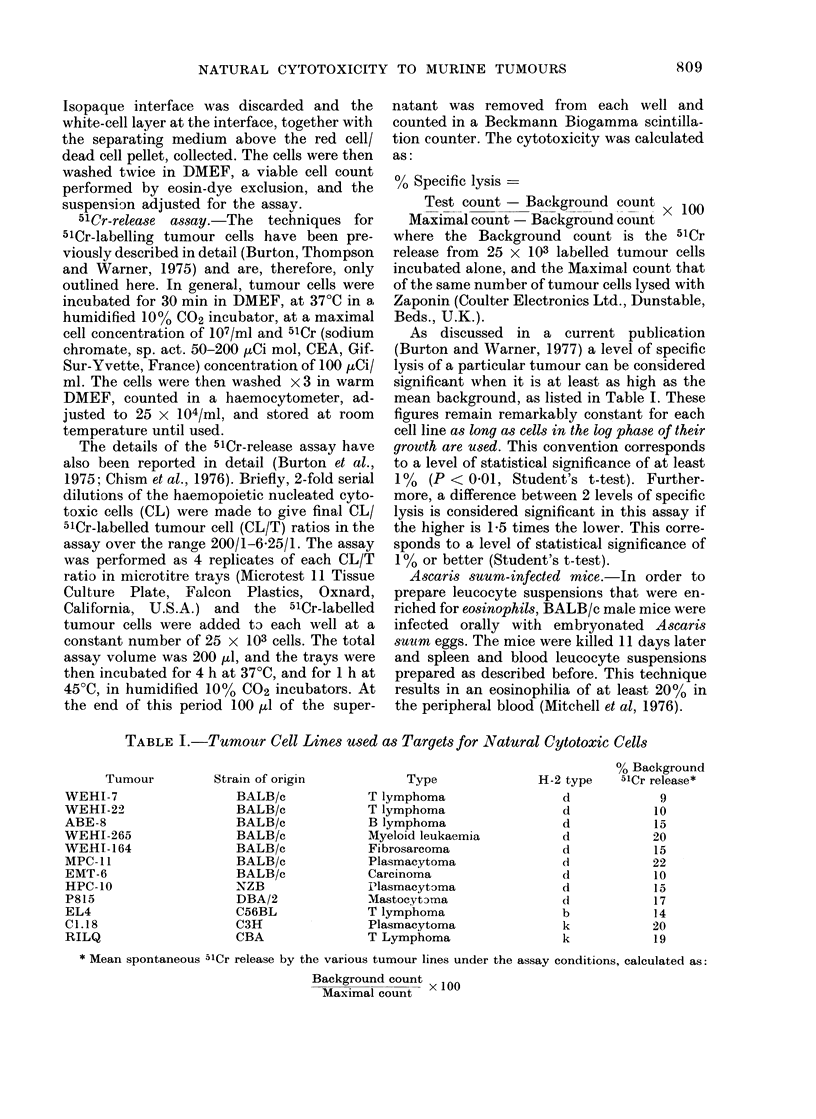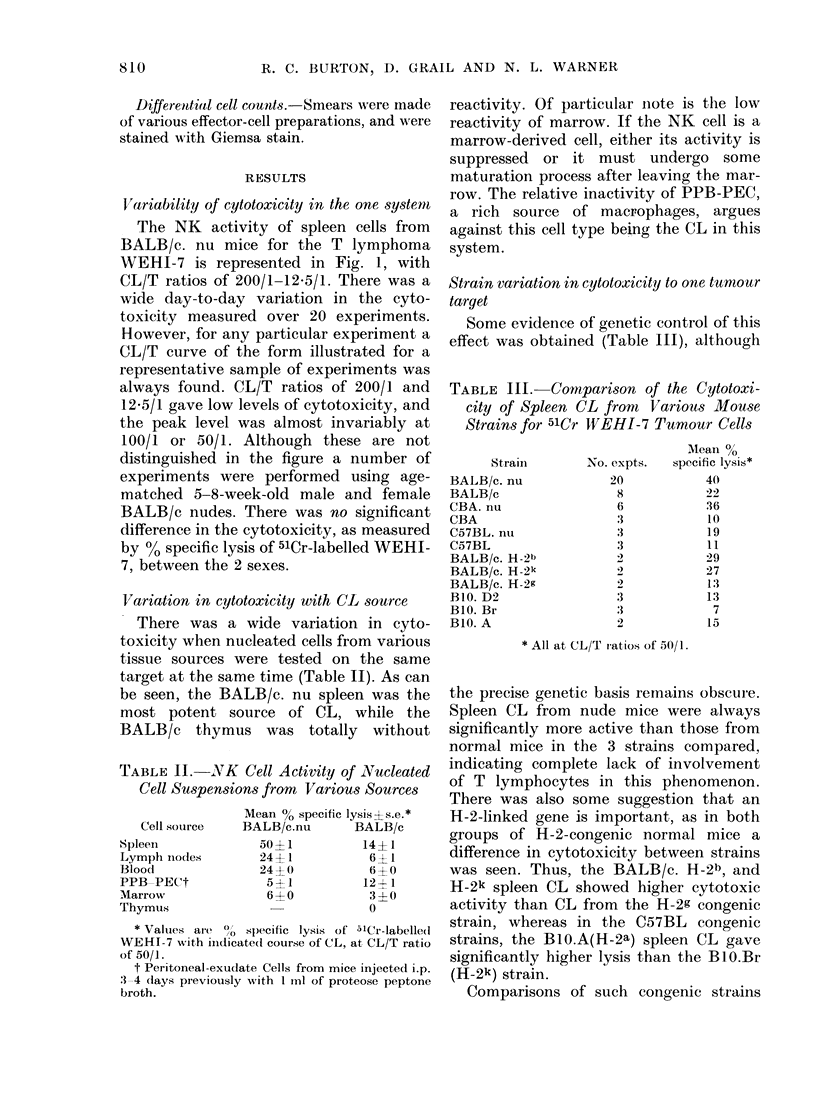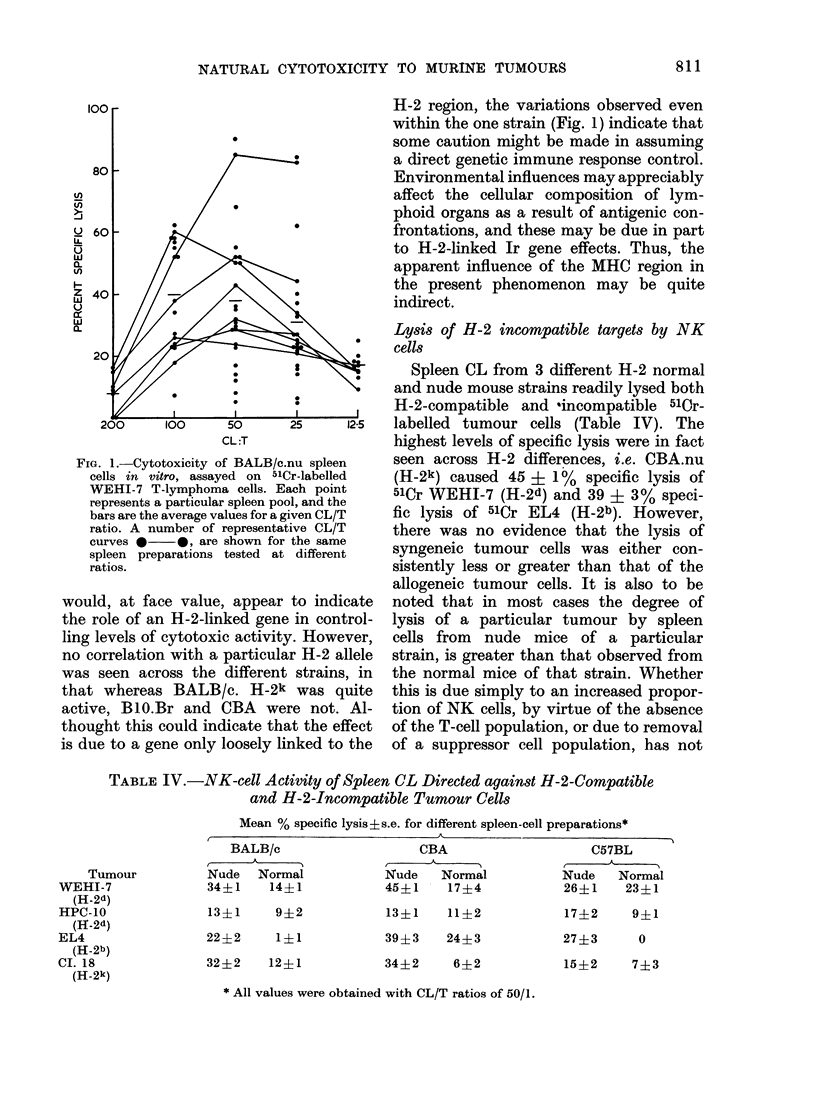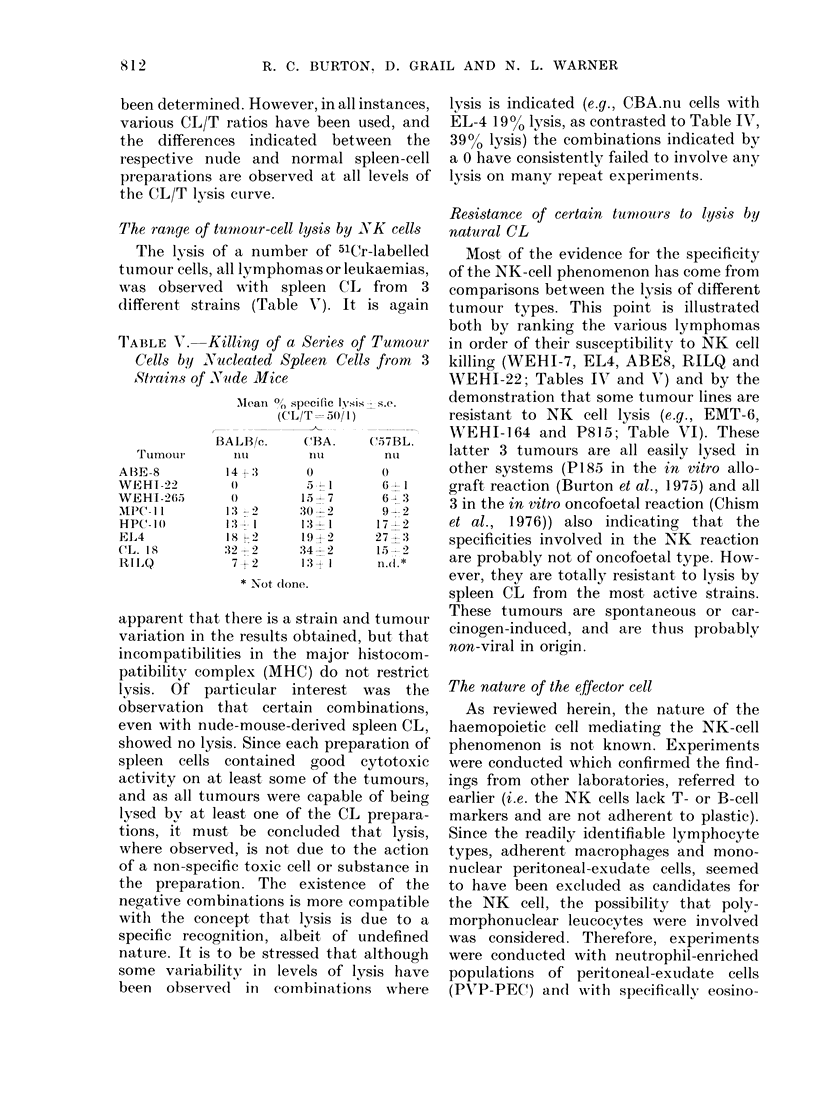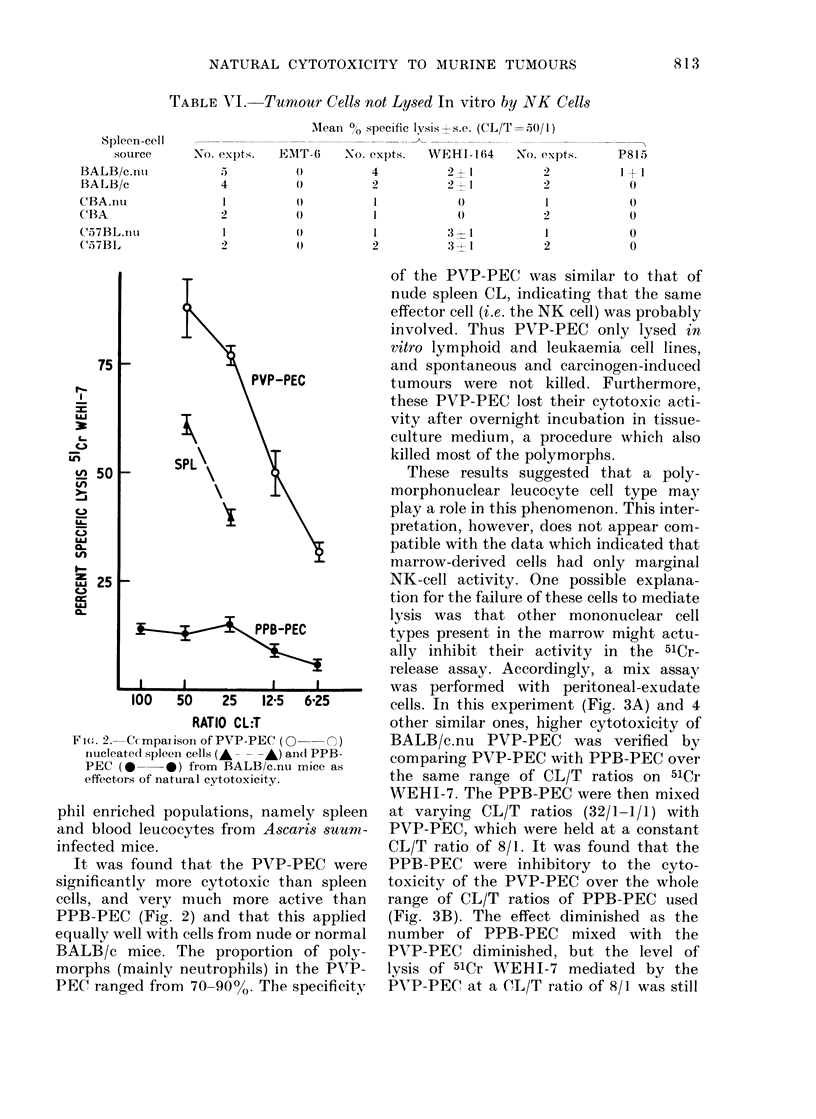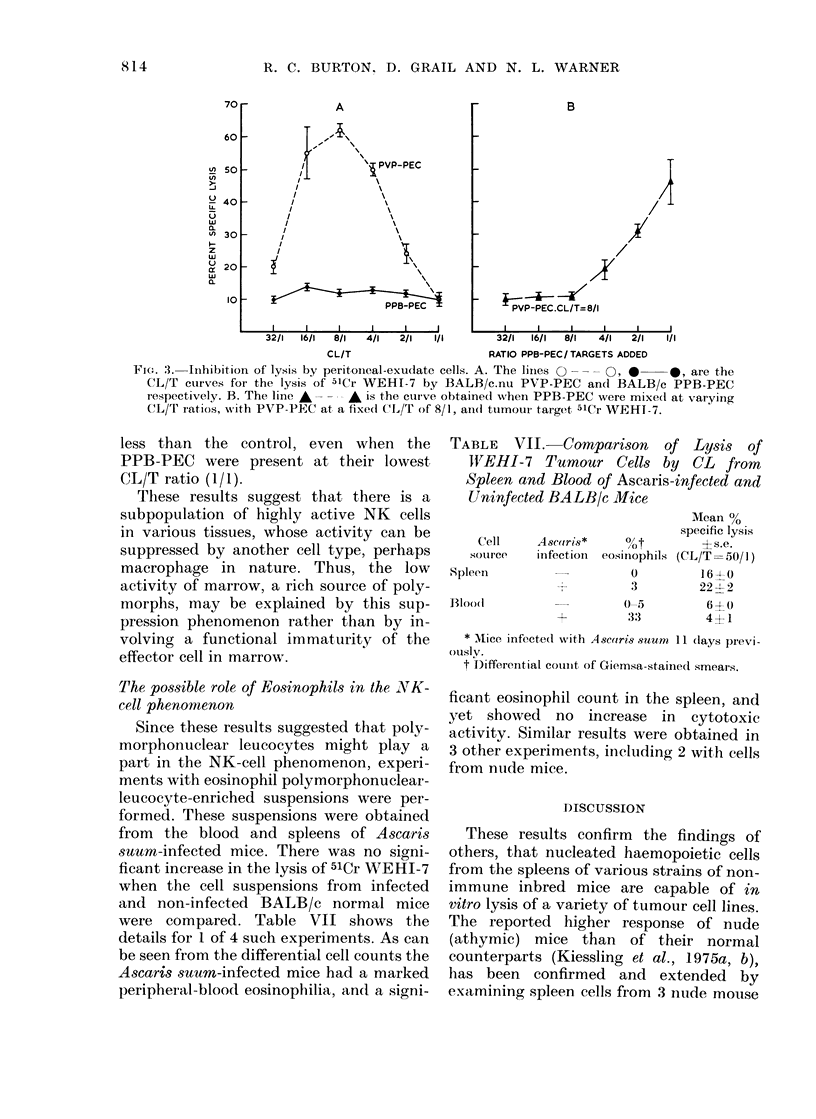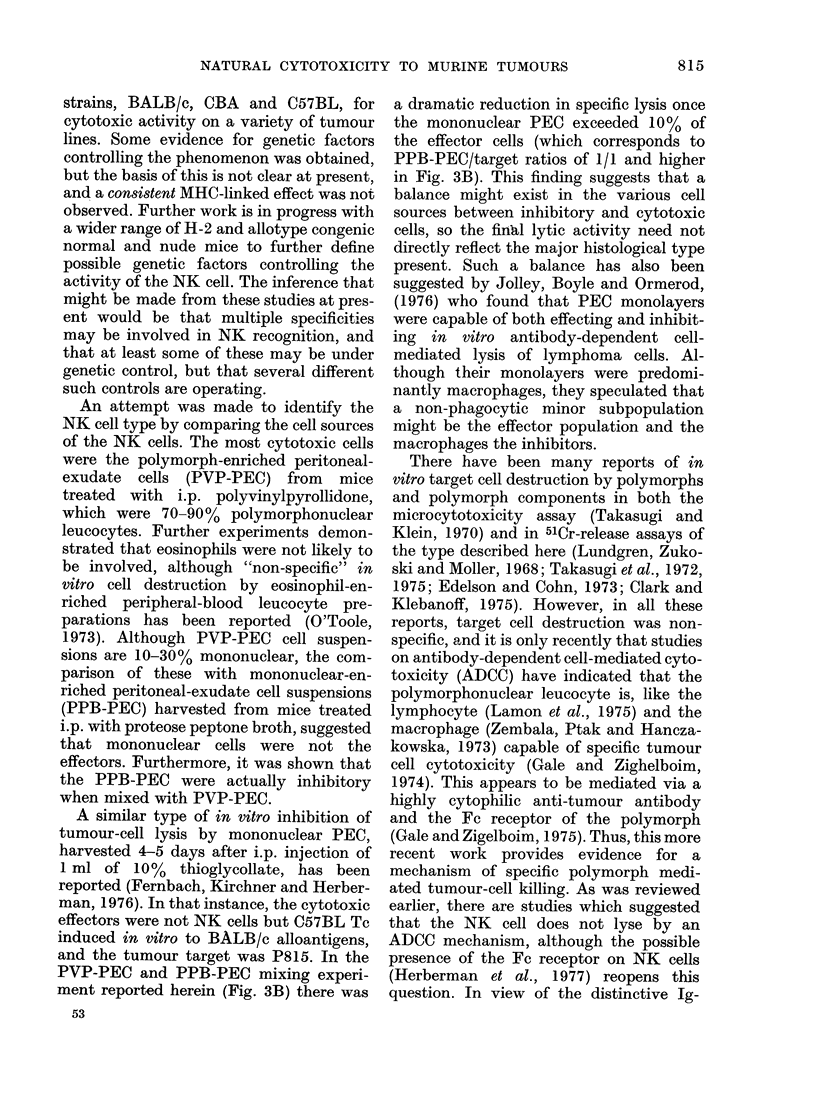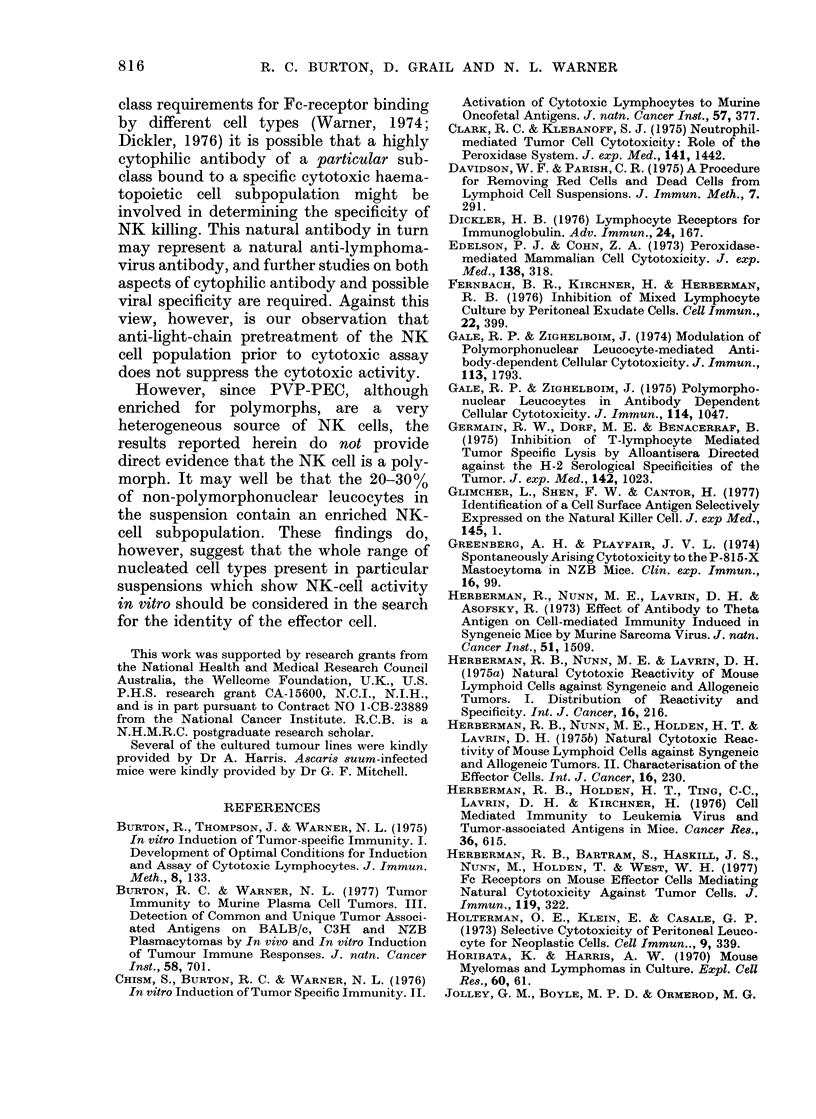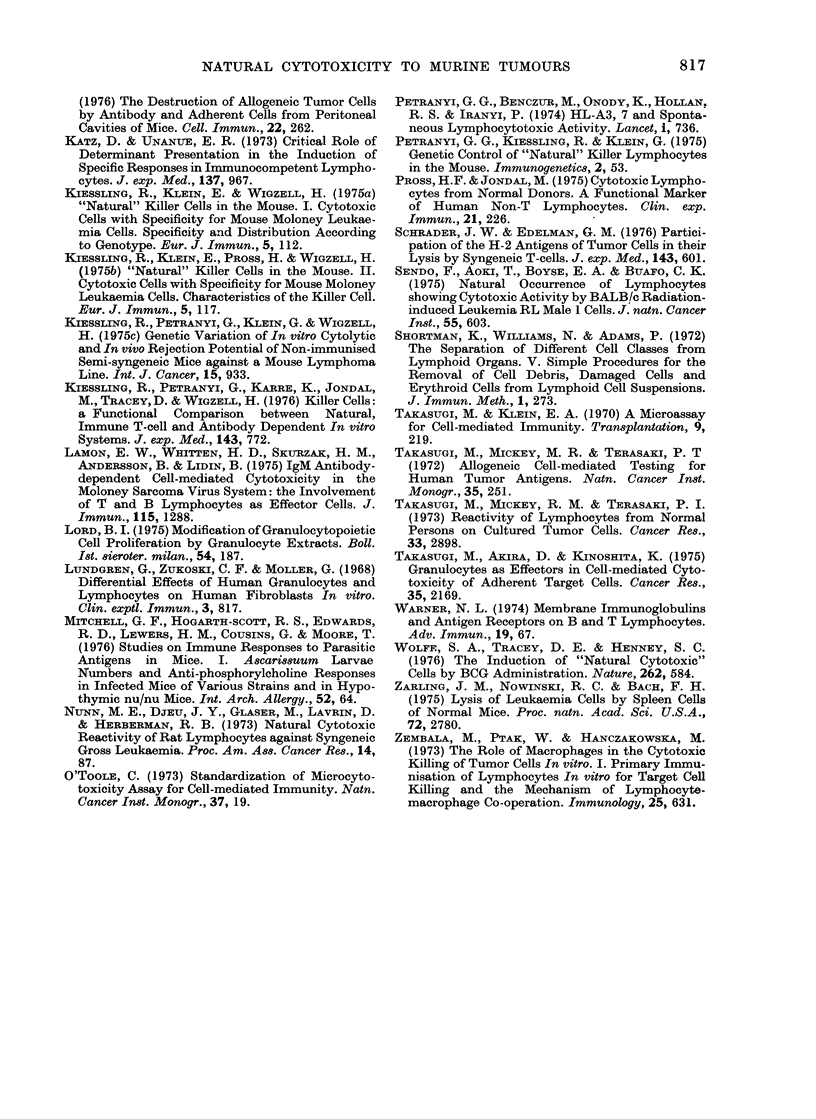# Natural cytotoxicity of haemopoietic cell populations against murine lymphoid tumours.

**DOI:** 10.1038/bjc.1978.119

**Published:** 1978-05

**Authors:** R. C. Burton, D. Grail, N. L. Warner

## Abstract

Homozygous nude and normal mice of 3 strains, BALB/c, CBA and C57BL, were used as sources of nucleated haemopoietic "natural killer" (NK) cells. These killer cells could lyse a wide range of syngeneic and allogeneic lymphoid tumour cell lines in vitro, and it was found that cell suspensions from nude mice were always significantly more active than those from normal mice, and that the most active effector population was a polymorph-enriched peritoneal-exudate cell suspension. Eosinophils did not appear to be involved in the phenomenon, and mononuclear peritoneal-exudate cell suspensions were actually highly inhibitory. Three non-lymphoid tumours, a carcinoma, a fibrosarcoma and a mastocytoma, were totally resistant to in vitro lysis. Although all susceptible tumour cell lines were C-type virus-associated, not all of these tumours were killed by all strain sources of spleen cells, indicating a specificity of killing.


*Br. J. Cancer* (1978) **37**, 806

### NATURAL CYTOTOXICITY OF HAEMOPOIETIC CELL POPULATIONS AGAINST MURINE LYMPHOID TUMOURS

R. C. BURTON, D. GRAIL AND N. L. WARNER*

*From the Genetics Unit, The Walter and Eliza Hall Institute of Medical Research, Melbourne, Australia*

Received 22 November 1977 Accepted 10 January 1978

**Summary.—Homozygous nude and normal mice of 3 strains, BALB/c, CBA and C57BL, were used as sources of nucleated haemopoietic "natural killer" (NK) cells. These killer cells could lyse a wide range of syngeneic and allogeneic lymphoid tumour cell lines *in vitro*, and it was found that cell suspensions from nude mice were always significantly more active than those from normal mice, and that the most active effector population was a polymorph-enriched peritoneal-exudate cell suspension. Eosinophils did not appear to be involved in the phenomenon, and mononuclear peritoneal-exudate cell suspensions were actually highly inhibitory. Three non-lymphoid tumours, a carcinoma, a fibrosarcoma and a mastocytoma, were totally resistant to *in vitro* lysis. Although all susceptible tumour cell lines were C-type virus-associated, not all of these tumours were killed by all strain sources of spleen cells, indicating a specificity of killing.**

REPORTS of the *in vitro* lysis of tumour cells by haemopoietic cells from unimmunized animals and humans have appeared sporadically in the literature. Cell-mediated cytotoxicity, as assayed by the lysis of tumour cell lines, has been reported in a number of different systems using human blood leucocytes (Takasugi, Mickey and Terasaki, 1973; Petranyi *et al.*, 1974), mouse spleen cells (Herberman *et al.*, 1973; Greenberg and Playfair, 1974) and rat spleen cells (Nunn *et al.*, 1973; Holterman, Klein and Casale, 1973) from normal donors. In 1975 reports from several laboratories clearly demonstrated the killing of various mouse lymphoma lines by spleen-cell suspensions from non-immune animals. Thus, Zarling, Nowinski and Bach (1975) drew attention to the fact that $^{51}$Cr release from a $^{51}$Cr-labelled AKR leukaemic cell line resulted from exposure to spleen cells from low, but not high, leukaemia strains of mice. Kiessling, Klein and Wigzell (1975*a*) demonstrated that murine spleen cells killed particular oncogenic-virus associated tumours *in vitro*, but did not lyse all such tumour cell lines, and this "specificity" of killing was subsequently confirmed by other workers (Herberman, Nunn and Lavrin, 1975*a*; Sendo *et al.*, 1975).

Current interest has focused on three aspects of this phenomenon: the identity of the "natural killer" (NK) cell, the genetic control of the phenomenon and the nature of the antigenic determinants recognized by the NK cells. To date, the NK cell has been characterized mainly in terms of negatives. Thus, it does not express markers of mature T or B cells (Kiessling *et al.*, 1975*b*; Herberman *et al.*, 1975*b*) nor the properties of "classical" adherent macrophages (Kiessling *et al.*, 1975*b*; Herberman *et al.*, 1975*b*). It does not manifest antibody-dependent cell-mediated cytotoxicity in the standard antibody-coated chicken-red-cell assay (Kiessling *et al.*, 1975*b*; 1976; Herberman

* Correspondence and reprint requests should be addressed to Dr Noel L. Warner, Department of Pathology, University of New Mexico School of Medicine, Albuquerque, New Mexico, 87131, U.S.A.

*et al.*, 1975*b*) although recent studies with a more sensitive system have suggested that the NK cell may express Fc receptors (Herberman *et al.*, 1977) which further emphasizes the need more strictly to compare these cells with K-cell function. To date, its positive qualities are a declining presence with increasing mouse age (Kiessling *et al.*, 1975*a*, *b*) a specific tissue distribution (Kiessling *et al.*, 1975*a*) an increased incidence in nude mice (Kiessling *et al.*, 1975*b*) and a lability in tissue culture at 37°C (Herberman *et al.*, 1975*b*). Recent studies have identified a specific cell-surface alloantigen on the NK cell (Glimcher, Shen and Cantor, 1977) and may provide an approach to its eventual isolation and precise characterization. Studies in a human system have indicated that peripheral human blood leucocytes with natural cytotoxic activity have complement receptors and are not T cells or B cells (Pross and Jondal, 1975); however, the murine natural killer cells lack complement receptors (Kiessling *et al.*, 1976).

Studies of the genetic control of this phenomenon have indicated that different mouse strains can manifest either high or low levels of "natural" cytotoxicity against particular tumour cell lines when their spleen cells are tested *in vitro* (Zarling *et al.*, 1975). More detailed analysis with congenic mouse lines and backcross analysis has indicated that the phenomenon is under polygenic control, at least one gene of which is linked to the major histocompatibility complex (MHC) (Petranyi, Kiessling and Klein, 1975; Kiessling *et al.*, 1975*c*), although our own studies described here do not demonstrate any evidence of MHC involvement in the responses studied.

This NK-cell phenomenon was first defined with a Rauscher-virus-induced leukaemia (Herberman *et al.*, 1973) and the possible specificity of NK cells for virally determined tumour-associated antigens (TAA) has been widely investigated. It is unlikely that histocompatibility antigens themselves are involved in the antigenic site, as has been suggested for TAAs in other systems (Germain, Dorf and Benacerraf, 1975; Schrader and Edelman, 1976) since $^{51}$Cr-labelled tumour target cells allogeneic to the NK cells are readily lysed (Zarling *et al.*, 1975; Kiessling *et al.*, 1975*a*) and unlabelled allogeneic tumour cells can also successfully compete in cellular competitive-inhibition assays with $^{51}$Cr-labelled tumour cells (Zarling *et al.*, 1975; Kiessling *et al.*, 1975*a*). Since normal lymphoid cells do not inhibit in such assays it has been inferred that normal tissue antigens are not involved (Zarling *et al.*, 1975). The viruses so far implicated belong to both the murine leukaemia virus (MuLV) and the Moloney sarcoma virus (MSV) groups (Herberman *et al.*, 1973; Zarling *et al.*, 1975; Herberman *et al.*, 1975*a*); however, no specific virion-or viral-cell-surface-associated antigens have yet been definitely designated as the determinants for NK cells. Comparison of the specificity of NK cells and cytotoxic T (Tc) cells from mice immune to MSV-induced tumours has indicated that different antigenic determinants are recognized by these different cell types (Herberman *et al.*, 1976).

In this paper we report our investigations into the nature and specificity of this phenomenon, with particular reference to a possible role for polymorphonuclear leucocytes in the *in vitro* lysis of $^{51}$Cr-labelled tumour cells.

### MATERIALS AND METHODS

*Tissue Culture Medium (DMEF)*.—Dulbecco's modified Eagles Medium (CSL, Melbourne, Australia) supplemented with 10% foetal calf serum (CSL, Melbourne, Australia) was used.

*Mice*.—Inbred mice obtained from the Hall Institute specific-pathogen-free colonies were: BALB/c An Bradley WEHI, BALB/c.nu (N8), C57BL/6J WEHI, CBA/CaH WEHI, CBA.nu (N10) and (BALB/c×C57BL) $F_1$ hybrids. Strains from the Hall Institute conventional colonies were: C57BL/6.nu (N10) (obtained from Dr I. Lefkovits, Basel, Switzerland) B10.D2/n Sn and B10.BR/Sg Sn

(obtained from Jackson Laboratories, U.S.A. in 1974) and BALB/c H-$2^b$, BALB/c H-$2^g$ and BALB/c H-$2^k$ (all 3 obtained in 1975 from Dr F. Lilly, New York, U.S.A.). All mice were 5–8-week-old males, unless otherwise indicated.

*Tumour cells.*—The tumour cell lines were propagated in continuous tissue culture as previously described (Horibata and Harris, 1970) and are listed in Table I. Their origins, with the exception of WEHI-265, have all been described in recent publications from this laboratory (Chism, Burton and Warner, 1976; Burton and Warner, 1977). WEHI-265 is a BALB/c tumour of the granulocyte–macrophage cell lineage and was induced by Abelson virus (Warner, N. L., Harris, A. W., Gutman, G., Metcalf, D., Burgess, A. W., Warr, G. W. and Haustein, D., manuscript in preparation).

*Cell suspensions.*—Spleen-cell suspensions were prepared by teasing and mincing spleens from mice killed by cervical dislocation through an 80 gauge stainless-steel sieve into ice-cold DMEF. After thoroughly pipetting the suspension, it was underlayered with FCS for 10 min in ice, to remove cell clumps and large debris. The supernatant was then transferred to another tube, underlayered again with FCS, and centrifuged at 1500 rev/min for 5 min, to pellet the cells and remove fine debris. The cells were then resuspended in 5 ml of 0·17M ammonium chloride, prewarmed to 37°C, to lyse the red cells. One min later a further 5 ml of ice-cold DMEF was added and the cells pelleted by a further light centrifugation. Finally, the cells were resuspended in ice-cold DMEF and stored on ice, while their viability was determined by an eosin-dye-exclusion count in a haemocytometer. The cell suspension was then adjusted as necessary for the assay. This technique is a modification of that described by Shortman, Williams and Adams (1972) and usually results in a nucleated erythrocyte-free spleen-cell suspension that is at least 80% viable.

*Lymph-node suspensions* were prepared from pooled mesenteric and peripheral lymph nodes in the same manner as for the spleen-cell suspensions, except for the omission of the ammonium chloride treatment.

*Thymus-cell suspensions* were prepared by treating thymus lobes in the same manner as lymph node cell suspensions.

*Marrow-cell suspensions* were prepared by aspirating the femoral marrow with a 21-g needle and suspending the cells in ice-cold DMEF by vigorous pipetting. The cell suspension was then treated in an identical manner to the spleen-cell suspension.

*Proteose-Peptone-Broth Peritoneal-Exudate Cells: PPB-PEC.*—These suspensions were prepared by injecting the appropriate mice i.p. with 1 ml of 10% proteose peptone broth (PPB) (Difco Laboratories, Detroit, Michigan) 3–4 days prior to harvest. The mice were killed by cervical dislocation and 3–5 ml of ice-cold 0·02 M phosphate-buffered saline (PBS) was injected into the peritoneal cavity. This was then aspirated with a pipette, and the cells pelleted by light centrifugation. The pellet was resuspended in 1–2 ml of ice-cold DMEF and a viability count made. This procedure, which is based on a previously described technique (Katz and Unanue, 1973) produces a cell suspension that is composed of at least 85% large mononuclear cells.

*Polyvinylpyrrolidone Peritoneal-exudate Cells: PVP-PEC.*—These suspensions were prepared by injecting 2 doses i.p. of 1 ml of 15% polyvinylpyrrolidone (PVP) (British Drug Houses, Melbourne, Australia, Stock 29579) in PBS 15 h and 2 h prior to harvest, in the appropriate mice. The cell suspension was then harvested and treated in the same way as for the PPB-PEC. The use of PVP in this way (Lord, 1975) produces a cell suspension which is usually at least 80% polymorphonuclear leucocytes (polymorphs). Considerable variation has been found between various batches of PVP in their ability to induce a primarily granulocytic exudate with cytotoxic activity, so that individual batches of PVP must be screened for this ability.

*Peripheral blood leucocytes.*—These were prepared according to a modification of the method of Davidson and Parish (1975). Briefly, the mice were exsanguinated by eye bleeding into tubes containing 0·5 ml of 0·8% sodium citrate solution as anti-coagulant. The blood was then divided into 5 ml aliquots, 20 $\mu$l of 25% sodium azide added (final concentration 0·1%), and the blood warmed to 20°C. It was then layered over 4·0 ml of Isopaque/Ficoll (prepared as described by Davidson and Parish, 1975) in U-bottomed glass siliconized centrifuge tubes. The tubes were placed in a centrifuge at room temperature and spun at 2000 $g$ for 15 min, with rapid acceleration to 2000 $g$ in 20 sec. After centrifugation the supernatant above the Ficoll/

Isopaque interface was discarded and the white-cell layer at the interface, together with the separating medium above the red cell/dead cell pellet, collected. The cells were then washed twice in DMEF, a viable cell count performed by eosin-dye exclusion, and the suspension adjusted for the assay.

*$^{51}$Cr-release assay.*—The techniques for $^{51}$Cr-labelling tumour cells have been previously described in detail (Burton, Thompson and Warner, 1975) and are, therefore, only outlined here. In general, tumour cells were incubated for 30 min in DMEF, at 37°C in a humidified 10% $CO_2$ incubator, at a maximal cell concentration of $10^7$/ml and $^{51}$Cr (sodium chromate, sp. act. 50–200 $\mu$Ci mol, CEA, Gif-Sur-Yvette, France) concentration of 100 $\mu$Ci/ml. The cells were then washed $\times 3$ in warm DMEF, counted in a haemocytometer, adjusted to $25 \times 10^4$/ml, and stored at room temperature until used.

The details of the $^{51}$Cr-release assay have also been reported in detail (Burton *et al.*, 1975; Chism *et al.*, 1976). Briefly, 2-fold serial dilutions of the haemopoietic nucleated cytotoxic cells (CL) were made to give final CL/$^{51}$Cr-labelled tumour cell (CL/T) ratios in the assay over the range 200/1–6·25/1. The assay was performed as 4 replicates of each CL/T ratio in microtitre trays (Microtest 11 Tissue Culture Plate, Falcon Plastics, Oxnard, California, U.S.A.) and the $^{51}$Cr-labelled tumour cells were added to each well at a constant number of $25 \times 10^3$ cells. The total assay volume was 200 $\mu$l, and the trays were then incubated for 4 h at 37°C, and for 1 h at 45°C, in humidified 10% $CO_2$ incubators. At the end of this period 100 $\mu$l of the supernatant was removed from each well and counted in a Beckmann Biogamma scintillation counter. The cytotoxicity was calculated as:

$$\% \text{ Specific lysis} = \frac{\text{Test count} - \text{Background count}}{\text{Maximal count} - \text{Background count}} \times 100$$

where the Background count is the $^{51}$Cr release from $25 \times 10^3$ labelled tumour cells incubated alone, and the Maximal count that of the same number of tumour cells lysed with Zaponin (Coulter Electronics Ltd., Dunstable, Beds., U.K.).

As discussed in a current publication (Burton and Warner, 1977) a level of specific lysis of a particular tumour can be considered significant when it is at least as high as the mean background, as listed in Table I. These figures remain remarkably constant for each cell line *as long as cells in the log phase of their growth are used.* This convention corresponds to a level of statistical significance of at least 1% ($P < 0{\cdot}01$, Student's t-test). Furthermore, a difference between 2 levels of specific lysis is considered significant in this assay if the higher is 1·5 times the lower. This corresponds to a level of statistical significance of 1% or better (Student's t-test).

*Ascaris suum-infected mice.*—In order to prepare leucocyte suspensions that were enriched for *eosinophils*, BALB/c male mice were infected orally with embryonated *Ascaris suum* eggs. The mice were killed 11 days later and spleen and blood leucocyte suspensions prepared as described before. This technique results in an eosinophilia of at least 20% in the peripheral blood (Mitchell *et al*, 1976).

TABLE I.—*Tumour Cell Lines used as Targets for Natural Cytotoxic Cells*

| Tumour | Strain of origin | Type | H-2 type | % Background $^{51}$Cr release* |
|---|---|---|---|---|
| WEHI-7 | BALB/c | T lymphoma | d | 9 |
| WEHI-22 | BALB/c | T lymphoma | d | 10 |
| ABE-8 | BALB/c | B lymphoma | d | 15 |
| WEHI-265 | BALB/c | Myeloid leukaemia | d | 20 |
| WEHI-164 | BALB/c | Fibrosarcoma | d | 15 |
| MPC-11 | BALB/c | Plasmacytoma | d | 22 |
| EMT-6 | BALB/c | Carcinoma | d | 10 |
| HPC-10 | NZB | Plasmacytoma | d | 15 |
| P815 | DBA/2 | Mastocytoma | d | 17 |
| EL4 | C56BL | T lymphoma | b | 14 |
| C1.18 | C3H | Plasmacytoma | k | 20 |
| RILQ | CBA | T Lymphoma | k | 19 |

* Mean spontaneous $^{51}$Cr release by the various tumour lines under the assay conditions, calculated as:

$$\frac{\text{Background count}}{\text{Maximal count}} \times 100$$

*Differential cell counts.*—Smears were made of various effector-cell preparations, and were stained with Giemsa stain.

RESULTS

*Variability of cytotoxicity in the one system*

The NK activity of spleen cells from BALB/c. nu mice for the T lymphoma WEHI-7 is represented in Fig. 1, with CL/T ratios of 200/1–12·5/1. There was a wide day-to-day variation in the cytotoxicity measured over 20 experiments. However, for any particular experiment a CL/T curve of the form illustrated for a representative sample of experiments was always found. CL/T ratios of 200/1 and 12·5/1 gave low levels of cytotoxicity, and the peak level was almost invariably at 100/1 or 50/1. Although these are not distinguished in the figure a number of experiments were performed using age-matched 5–8-week-old male and female BALB/c nudes. There was *no* significant difference in the cytotoxicity, as measured by % specific lysis of $^{51}$Cr-labelled WEHI-7, between the 2 sexes.

*Variation in cytotoxicity with CL source*

There was a wide variation in cytotoxicity when nucleated cells from various tissue sources were tested on the same target at the same time (Table II). As can be seen, the BALB/c. nu spleen was the most potent source of CL, while the BALB/c thymus was totally without reactivity. Of particular note is the low reactivity of marrow. If the NK cell is a marrow-derived cell, either its activity is suppressed or it must undergo some maturation process after leaving the marrow. The relative inactivity of PPB-PEC, a rich source of macrophages, argues against this cell type being the CL in this system.

TABLE II.—*NK Cell Activity of Nucleated Cell Suspensions from Various Sources*

| Cell source | Mean % specific lysis ± s.e.* BALB/c.nu | BALB/c |
|---|---|---|
| Spleen | 50 ± 1 | 14 ± 1 |
| Lymph nodes | 24 ± 1 | 6 ± 1 |
| Blood | 24 ± 0 | 6 ± 0 |
| PPB-PEC† | 5 ± 1 | 12 ± 1 |
| Marrow | 6 ± 0 | 3 ± 0 |
| Thymus | — | 0 |

* Values are % specific lysis of $^{51}$Cr-labelled WEHI-7 with indicated course of CL, at CL/T ratio of 50/1.

† Peritoneal-exudate Cells from mice injected i.p. 3–4 days previously with 1 ml of proteose peptone broth.

*Strain variation in cytotoxicity to one tumour target*

Some evidence of genetic control of this effect was obtained (Table III), although the precise genetic basis remains obscure. Spleen CL from nude mice were always significantly more active than those from normal mice in the 3 strains compared, indicating complete lack of involvement of T lymphocytes in this phenomenon. There was also some suggestion that an H-2-linked gene is important, as in both groups of H-2-congenic normal mice a difference in cytotoxicity between strains was seen. Thus, the BALB/c. H-2$^{b}$, and H-2$^{k}$ spleen CL showed higher cytotoxic activity than CL from the H-2$^{g}$ congenic strain, whereas in the C57BL congenic strains, the B10.A(H-2$^{a}$) spleen CL gave significantly higher lysis than the B10.Br (H-2$^{k}$) strain.

Comparisons of such congenic strains

TABLE III.—*Comparison of the Cytotoxicity of Spleen CL from Various Mouse Strains for $^{51}$Cr WEHI-7 Tumour Cells*

| Strain | No. expts. | Mean % specific lysis* |
|---|---|---|
| BALB/c. nu | 20 | 40 |
| BALB/c | 8 | 22 |
| CBA. nu | 6 | 36 |
| CBA | 3 | 10 |
| C57BL. nu | 3 | 19 |
| C57BL | 3 | 11 |
| BALB/c. H-2$^{b}$ | 2 | 29 |
| BALB/c. H-2$^{k}$ | 2 | 27 |
| BALB/c. H-2$^{g}$ | 2 | 13 |
| B10. D2 | 3 | 13 |
| B10. Br | 3 | 7 |
| B10. A | 2 | 15 |

* All at CL/T ratios of 50/1.

FIG. 1.—Cytotoxicity of BALB/c.nu spleen cells *in vitro*, assayed on $^{51}$Cr-labelled WEHI-7 T-lymphoma cells. Each point represents a particular spleen pool, and the bars are the average values for a given CL/T ratio. A number of representative CL/T curves ●——●, are shown for the same spleen preparations tested at different ratios.

would, at face value, appear to indicate the role of an H-2-linked gene in controlling levels of cytotoxic activity. However, no correlation with a particular H-2 allele was seen across the different strains, in that whereas BALB/c. H-$2^k$ was quite active, B10.Br and CBA were not. Althought this could indicate that the effect is due to a gene only loosely linked to the H-2 region, the variations observed even within the one strain (Fig. 1) indicate that some caution might be made in assuming a direct genetic immune response control. Environmental influences may appreciably affect the cellular composition of lymphoid organs as a result of antigenic confrontations, and these may be due in part to H-2-linked Ir gene effects. Thus, the apparent influence of the MHC region in the present phenomenon may be quite indirect.

*Lysis of H-2 incompatible targets by NK cells*

Spleen CL from 3 different H-2 normal and nude mouse strains readily lysed both H-2-compatible and incompatible $^{51}$Cr-labelled tumour cells (Table IV). The highest levels of specific lysis were in fact seen across H-2 differences, *i.e.* CBA.nu (H-$2^k$) caused $45 \pm 1\%$ specific lysis of $^{51}$Cr WEHI-7 (H-$2^d$) and $39 \pm 3\%$ specific lysis of $^{51}$Cr EL4 (H-$2^b$). However, there was no evidence that the lysis of syngeneic tumour cells was either consistently less or greater than that of the allogeneic tumour cells. It is also to be noted that in most cases the degree of lysis of a particular tumour by spleen cells from nude mice of a particular strain, is greater than that observed from the normal mice of that strain. Whether this is due simply to an increased proportion of NK cells, by virtue of the absence of the T-cell population, or due to removal of a suppressor cell population, has not

TABLE IV.—*NK-cell Activity of Spleen CL Directed against H-2-Compatible and H-2-Incompatible Tumour Cells*

| | Mean % specific lysis ± s.e. for different spleen-cell preparations* | | | | | |
|---|---|---|---|---|---|---|
| | BALB/c | | CBA | | C57BL | |
| Tumour | Nude | Normal | Nude | Normal | Nude | Normal |
| WEHI-7 (H-$2^d$) | $34 \pm 1$ | $14 \pm 1$ | $45 \pm 1$ | $17 \pm 4$ | $26 \pm 1$ | $23 \pm 1$ |
| HPC-10 (H-$2^d$) | $13 \pm 1$ | $9 \pm 2$ | $13 \pm 1$ | $11 \pm 2$ | $17 \pm 2$ | $9 \pm 1$ |
| EL4 (H-$2^b$) | $22 \pm 2$ | $1 \pm 1$ | $39 \pm 3$ | $24 \pm 3$ | $27 \pm 3$ | 0 |
| CI. 18 (H-$2^k$) | $32 \pm 2$ | $12 \pm 1$ | $34 \pm 2$ | $6 \pm 2$ | $15 \pm 2$ | $7 \pm 3$ |

* All values were obtained with CL/T ratios of 50/1.

been determined. However, in all instances, various CL/T ratios have been used, and the differences indicated between the respective nude and normal spleen-cell preparations are observed at all levels of the CL/T lysis curve.

*The range of tumour-cell lysis by NK cells*

The lysis of a number of $^{51}$Cr-labelled tumour cells, all lymphomas or leukaemias, was observed with spleen CL from 3 different strains (Table V). It is again

TABLE V.—*Killing of a Series of Tumour Cells by Nucleated Spleen Cells from 3 Strains of Nude Mice*

| Tumour | Mean % specific lysis ± s.e. (CL/T = 50/1) BALB/c. nu | CBA. nu | C57BL. nu |
|---|---|---|---|
| ABE-8 | 14 ± 3 | 0 | 0 |
| WEHI-22 | 0 | 5 ± 1 | 6 ± 1 |
| WEHI-265 | 0 | 15 ± 7 | 6 ± 3 |
| MPC-11 | 13 ± 2 | 30 ± 2 | 9 ± 2 |
| HPC-10 | 13 ± 1 | 13 ± 1 | 17 ± 2 |
| EL4 | 18 ± 2 | 19 ± 2 | 27 ± 3 |
| CL. 18 | 32 ± 2 | 34 ± 2 | 15 ± 2 |
| RILQ | 7 ± 2 | 13 ± 1 | n.d.* |

* Not done.

apparent that there is a strain and tumour variation in the results obtained, but that incompatibilities in the major histocompatibility complex (MHC) do not restrict lysis. Of particular interest was the observation that certain combinations, even with nude-mouse-derived spleen CL, showed no lysis. Since each preparation of spleen cells contained good cytotoxic activity on at least some of the tumours, and as all tumours were capable of being lysed by at least one of the CL preparations, it must be concluded that lysis, where observed, is not due to the action of a non-specific toxic cell or substance in the preparation. The existence of the negative combinations is more compatible with the concept that lysis is due to a specific recognition, albeit of undefined nature. It is to be stressed that although some variability in levels of lysis have been observed in combinations where lysis is indicated (*e.g.*, CBA.nu cells with EL-4 19% lysis, as contrasted to Table IV, 39% lysis) the combinations indicated by a 0 have consistently failed to involve any lysis on many repeat experiments.

*Resistance of certain tumours to lysis by natural CL*

Most of the evidence for the specificity of the NK-cell phenomenon has come from comparisons between the lysis of different tumour types. This point is illustrated both by ranking the various lymphomas in order of their susceptibility to NK cell killing (WEHI-7, EL4, ABE8, RILQ and WEHI-22; Tables IV and V) and by the demonstration that some tumour lines are resistant to NK cell lysis (*e.g.*, EMT-6, WEHI-164 and P815; Table VI). These latter 3 tumours are all easily lysed in other systems (P185 in the *in vitro* allograft reaction (Burton *et al.*, 1975) and all 3 in the *in vitro* oncofoetal reaction (Chism *et al.*, 1976)) also indicating that the specificities involved in the NK reaction are probably not of oncofoetal type. However, they are totally resistant to lysis by spleen CL from the most active strains. These tumours are spontaneous or carcinogen-induced, and are thus probably *non*-viral in origin.

*The nature of the effector cell*

As reviewed herein, the nature of the haemopoietic cell mediating the NK-cell phenomenon is not known. Experiments were conducted which confirmed the findings from other laboratories, referred to earlier (*i.e.* the NK cells lack T- or B-cell markers and are not adherent to plastic). Since the readily identifiable lymphocyte types, adherent macrophages and mononuclear peritoneal-exudate cells, seemed to have been excluded as candidates for the NK cell, the possibility that polymorphonuclear leucocytes were involved was considered. Therefore, experiments were conducted with neutrophil-enriched populations of peritoneal-exudate cells (PVP-PEC) and with specifically eosino-

TABLE VI.—*Tumour Cells not Lysed* In vitro *by NK Cells*

| Spleen-cell source | Mean % specific lysis±s.e. (CL/T=50/1) | | | | | |
|---|---|---|---|---|---|---|
| | No. expts. | EMT-6 | No. expts. | WEHI-164 | No. expts. | P815 |
| BALB/c.nu | 5 | 0 | 4 | 2±1 | 2 | 1±1 |
| BALB/c | 4 | 0 | 2 | 2±1 | 2 | 0 |
| CBA.nu | 1 | 0 | 1 | 0 | 1 | 0 |
| CBA | 2 | 0 | 1 | 0 | 2 | 0 |
| C57BL.nu | 1 | 0 | 1 | 3±1 | 1 | 0 |
| C57BL | 2 | 0 | 2 | 3±1 | 2 | 0 |

FIG. 2.—Comparison of PVP-PEC (○——○) nucleated spleen cells (▲- - -▲) and PPB-PEC (●——●) from BALB/c.nu mice as effectors of natural cytotoxicity.

phil enriched populations, namely spleen and blood leucocytes from *Ascaris suum*-infected mice.

It was found that the PVP-PEC were significantly more cytotoxic than spleen cells, and very much more active than PPB-PEC (Fig. 2) and that this applied equally well with cells from nude or normal BALB/c mice. The proportion of polymorphs (mainly neutrophils) in the PVP-PEC ranged from 70–90%. The specificity of the PVP-PEC was similar to that of nude spleen CL, indicating that the same effector cell (*i.e.* the NK cell) was probably involved. Thus PVP-PEC only lysed *in vitro* lymphoid and leukaemia cell lines, and spontaneous and carcinogen-induced tumours were not killed. Furthermore, these PVP-PEC lost their cytotoxic activity after overnight incubation in tissue-culture medium, a procedure which also killed most of the polymorphs.

These results suggested that a polymorphonuclear leucocyte cell type may play a role in this phenomenon. This interpretation, however, does not appear compatible with the data which indicated that marrow-derived cells had only marginal NK-cell activity. One possible explanation for the failure of these cells to mediate lysis was that other mononuclear cell types present in the marrow might actually inhibit their activity in the $^{51}$Cr-release assay. Accordingly, a mix assay was performed with peritoneal-exudate cells. In this experiment (Fig. 3A) and 4 other similar ones, higher cytotoxicity of BALB/c.nu PVP-PEC was verified by comparing PVP-PEC with PPB-PEC over the same range of CL/T ratios on $^{51}$Cr WEHI-7. The PPB-PEC were then mixed at varying CL/T ratios (32/1–1/1) with PVP-PEC, which were held at a constant CL/T ratio of 8/1. It was found that the PPB-PEC were inhibitory to the cytotoxicity of the PVP-PEC over the whole range of CL/T ratios of PPB-PEC used (Fig. 3B). The effect diminished as the number of PPB-PEC mixed with the PVP-PEC diminished, but the level of lysis of $^{51}$Cr WEHI-7 mediated by the PVP-PEC at a CL/T ratio of 8/1 was still

FIG. 3.—Inhibition of lysis by peritoneal-exudate cells. A. The lines ○- - - -○, ●——●, are the CL/T curves for the lysis of $^{51}$Cr WEHI-7 by BALB/c.nu PVP-PEC and BALB/c PPB-PEC respectively. B. The line ▲- - - -▲ is the curve obtained when PPB-PEC were mixed at varying CL/T ratios, with PVP-PEC at a fixed CL/T of 8/1, and tumour target $^{51}$Cr WEHI-7.

less than the control, even when the PPB-PEC were present at their lowest CL/T ratio (1/1).

These results suggest that there is a subpopulation of highly active NK cells in various tissues, whose activity can be suppressed by another cell type, perhaps macrophage in nature. Thus, the low activity of marrow, a rich source of polymorphs, may be explained by this suppression phenomenon rather than by involving a functional immaturity of the effector cell in marrow.

### *The possible role of Eosinophils in the NK-cell phenomenon*

Since these results suggested that polymorphonuclear leucocytes might play a part in the NK-cell phenomenon, experiments with eosinophil polymorphonuclear-leucocyte-enriched suspensions were performed. These suspensions were obtained from the blood and spleens of *Ascaris suum*-infected mice. There was no significant increase in the lysis of $^{51}$Cr WEHI-7 when the cell suspensions from infected and non-infected BALB/c normal mice were compared. Table VII shows the details for 1 of 4 such experiments. As can be seen from the differential cell counts the *Ascaris suum*-infected mice had a marked peripheral-blood eosinophilia, and a significant eosinophil count in the spleen, and yet showed no increase in cytotoxic activity. Similar results were obtained in 3 other experiments, including 2 with cells from nude mice.

TABLE VII.—*Comparison of Lysis of WEHI-7 Tumour Cells by CL from Spleen and Blood of* Ascaris-*infected and Uninfected BALB/c Mice*

| Cell source | *Ascaris** infection | %† eosinophils | Mean % specific lysis ±s.e. (CL/T=50/1) |
|---|---|---|---|
| Spleen | — | 0 | 16±0 |
| | + | 3 | 22±2 |
| Blood | — | 0–5 | 6±0 |
| | + | 33 | 4±1 |

* Mice infected with *Ascaris suum* 11 days previously.

† Differential count of Giemsa-stained smears.

### DISCUSSION

These results confirm the findings of others, that nucleated haemopoietic cells from the spleens of various strains of non-immune inbred mice are capable of *in vitro* lysis of a variety of tumour cell lines. The reported higher response of nude (athymic) mice than of their normal counterparts (Kiessling *et al.*, 1975*a*, *b*), has been confirmed and extended by examining spleen cells from 3 nude mouse strains, BALB/c, CBA and C57BL, for cytotoxic activity on a variety of tumour lines. Some evidence for genetic factors controlling the phenomenon was obtained, but the basis of this is not clear at present, and a *consistent* MHC-linked effect was not observed. Further work is in progress with a wider range of H-2 and allotype congenic normal and nude mice to further define possible genetic factors controlling the activity of the NK cell. The inference that might be made from these studies at present would be that multiple specificities may be involved in NK recognition, and that at least some of these may be under genetic control, but that several different such controls are operating.

An attempt was made to identify the NK cell type by comparing the cell sources of the NK cells. The most cytotoxic cells were the polymorph-enriched peritoneal-exudate cells (PVP-PEC) from mice treated with i.p. polyvinylpyrollidone, which were 70–90% polymorphonuclear leucocytes. Further experiments demonstrated that eosinophils were not likely to be involved, although "non-specific" *in vitro* cell destruction by eosinophil-enriched peripheral-blood leucocyte preparations has been reported (O'Toole, 1973). Although PVP-PEC cell suspensions are 10–30% mononuclear, the comparison of these with mononuclear-enriched peritoneal-exudate cell suspensions (PPB-PEC) harvested from mice treated i.p. with proteose peptone broth, suggested that mononuclear cells were not the effectors. Furthermore, it was shown that the PPB-PEC were actually inhibitory when mixed with PVP-PEC.

A similar type of *in vitro* inhibition of tumour-cell lysis by mononuclear PEC, harvested 4–5 days after i.p. injection of 1 ml of 10% thioglycollate, has been reported (Fernbach, Kirchner and Herberman, 1976). In that instance, the cytotoxic effectors were not NK cells but C57BL Tc induced *in vitro* to BALB/c alloantigens, and the tumour target was P815. In the PVP-PEC and PPB-PEC mixing experiment reported herein (Fig. 3B) there was a dramatic reduction in specific lysis once the mononuclear PEC exceeded 10% of the effector cells (which corresponds to PPB-PEC/target ratios of 1/1 and higher in Fig. 3B). This finding suggests that a balance might exist in the various cell sources between inhibitory and cytotoxic cells, so the final lytic activity need not directly reflect the major histological type present. Such a balance has also been suggested by Jolley, Boyle and Ormerod, (1976) who found that PEC monolayers were capable of both effecting and inhibiting *in vitro* antibody-dependent cell-mediated lysis of lymphoma cells. Although their monolayers were predominantly macrophages, they speculated that a non-phagocytic minor subpopulation might be the effector population and the macrophages the inhibitors.

There have been many reports of *in vitro* target cell destruction by polymorphs and polymorph components in both the microcytotoxicity assay (Takasugi and Klein, 1970) and in $^{51}$Cr-release assays of the type described here (Lundgren, Zukoski and Moller, 1968; Takasugi *et al.*, 1972, 1975; Edelson and Cohn, 1973; Clark and Klebanoff, 1975). However, in all these reports, target cell destruction was non-specific, and it is only recently that studies on antibody-dependent cell-mediated cytotoxicity (ADCC) have indicated that the polymorphonuclear leucocyte is, like the lymphocyte (Lamon *et al.*, 1975) and the macrophage (Zembala, Ptak and Hanczakowska, 1973) capable of specific tumour cell cytotoxicity (Gale and Zighelboim, 1974). This appears to be mediated via a highly cytophilic anti-tumour antibody and the Fc receptor of the polymorph (Gale and Zigelboim, 1975). Thus, this more recent work provides evidence for a mechanism of specific polymorph mediated tumour-cell killing. As was reviewed earlier, there are studies which suggested that the NK cell does not lyse by an ADCC mechanism, although the possible presence of the Fc receptor on NK cells (Herberman *et al.*, 1977) reopens this question. In view of the distinctive Igclass requirements for Fc-receptor binding by different cell types (Warner, 1974; Dickler, 1976) it is possible that a highly cytophilic antibody of a *particular* subclass bound to a specific cytotoxic haematopoietic cell subpopulation might be involved in determining the specificity of NK killing. This natural antibody in turn may represent a natural anti-lymphoma-virus antibody, and further studies on both aspects of cytophilic antibody and possible viral specificity are required. Against this view, however, is our observation that anti-light-chain pretreatment of the NK cell population prior to cytotoxic assay does not suppress the cytotoxic activity.

However, since PVP-PEC, although enriched for polymorphs, are a very heterogeneous source of NK cells, the results reported herein do *not* provide direct evidence that the NK cell is a polymorph. It may well be that the 20–30% of non-polymorphonuclear leucocytes in the suspension contain an enriched NK-cell subpopulation. These findings do, however, suggest that the whole range of nucleated cell types present in particular suspensions which show NK-cell activity *in vitro* should be considered in the search for the identity of the effector cell.

This work was supported by research grants from the National Health and Medical Research Council Australia, the Wellcome Foundation, U.K., U.S. P.H.S. research grant CA-15600, N.C.I., N.I.H., and is in part pursuant to Contract NO 1-CB-23889 from the National Cancer Institute. R.C.B. is a N.H.M.R.C. postgraduate research scholar.

Several of the cultured tumour lines were kindly provided by Dr A. Harris. *Ascaris suum*-infected mice were kindly provided by Dr G. F. Mitchell.

### REFERENCES

Burton, R., Thompson, J. & Warner, N. L. (1975) *In vitro* Induction of Tumor-specific Immunity. I. Development of Optimal Conditions for Induction and Assay of Cytotoxic Lymphocytes. *J. Immun. Meth.*, **8**, 133.

Burton, R. C. & Warner, N. L. (1977) Tumor Immunity to Murine Plasma Cell Tumors. III. Detection of Common and Unique Tumor Associated Antigens on BALB/c, C3H and NZB Plasmacytomas by *In vivo* and *In vitro* Induction of Tumour Immune Responses. *J. natn. Cancer Inst.*, **58**, 701.

Chism, S., Burton, R. C. & Warner, N. L. (1976) *In vitro* Induction of Tumor Specific Immunity. II. Activation of Cytotoxic Lymphocytes to Murine Oncofetal Antigens. *J. natn. Cancer Inst.*, **57**, 377.

Clark, R. C. & Klebanoff, S. J. (1975) Neutrophil-mediated Tumor Cell Cytotoxicity: Role of the Peroxidase System. *J. exp. Med.*, **141**, 1442.

Davidson, W. F. & Parish, C. R. (1975) A Procedure for Removing Red Cells and Dead Cells from Lymphoid Cell Suspensions. *J. Immun. Meth.*, **7**. 291.

Dickler, H. B. (1976) Lymphocyte Receptors for Immunoglobulin. *Adv. Immun.*, **24**, 167.

Edelson, P. J. & Cohn, Z. A. (1973) Peroxidase-mediated Mammalian Cell Cytotoxicity. *J. exp. Med.*, **138**, 318.

Fernbach, B. R., Kirchner, H. & Herberman, R. B. (1976) Inhibition of Mixed Lymphocyte Culture by Peritoneal Exudate Cells. *Cell Immun.*, **22**, 399.

Gale, R. P. & Zighelboim, J. (1974) Modulation of Polymorphonuclear Leucocyte-mediated Antibody-dependent Cellular Cytotoxicity. *J. Immun.*, **113**, 1793.

Gale, R. P. & Zighelboim, J. (1975) Polymorphonuclear Leucocytes in Antibody Dependent Cellular Cytotoxicity. *J. Immun.*, **114**, 1047.

Germain, R. W., Dorf, M. E. & Benacerraf, B. (1975) Inhibition of T-lymphocyte Mediated Tumor Specific Lysis by Alloantisera Directed against the H-2 Serological Specificities of the Tumor. *J. exp. Med.*, **142**, 1023.

Glimcher, L., Shen, F. W. & Cantor, H. (1977) Identification of a Cell Surface Antigen Selectively Expressed on the Natural Killer Cell. *J. exp Med.*, **145**, 1.

Greenberg, A. H. & Playfair, J. V. L. (1974) Spontaneously Arising Cytotoxicity to the P-815-X Mastocytoma in NZB Mice. *Clin. exp. Immun.*, **16**, 99.

Herberman, R., Nunn, M. E., Lavrin, D. H. & Asofsky, R. (1973) Effect of Antibody to Theta Antigen on Cell-mediated Immunity Induced in Syngeneic Mice by Murine Sarcoma Virus. *J. natn. Cancer Inst.*, **51**, 1509.

Herberman, R. B., Nunn, M. E. & Lavrin, D. H. (1975*a*) Natural Cytotoxic Reactivity of Mouse Lymphoid Cells against Syngeneic and Allogeneic Tumors. I. Distribution of Reactivity and Specificity. *Int. J. Cancer*, **16**, 216.

Herberman, R. B., Nunn, M. E., Holden, H. T. & Lavrin, D. H. (1975*b*) Natural Cytotoxic Reactivity of Mouse Lymphoid Cells against Syngeneic and Allogeneic Tumors. II. Characterisation of the Effector Cells. *Int. J. Cancer*, **16**, 230.

Herberman, R. B., Holden, H. T., Ting, C-C., Lavrin, D. H. & Kirchner, H. (1976) Cell Mediated Immunity to Leukemia Virus and Tumor-associated Antigens in Mice. *Cancer Res.*, **36**, 615.

Herberman, R. B., Bartram, S., Haskill, J. S., Nunn, M., Holden, T. & West, W. H. (1977) Fc Receptors on Mouse Effector Cells Mediating Natural Cytotoxicity Against Tumor Cells. *J. Immun.*, **119**, 322.

Holterman, O. E., Klein, E. & Casale, G. P. (1973) Selective Cytotoxicity of Peritoneal Leucocyte for Neoplastic Cells. *Cell Immun.*, **9**, 339.

Horibata, K. & Harris, A. W. (1970) Mouse Myelomas and Lymphomas in Culture. *Expl. Cell Res.*, **60**, 61.

Jolley, G. M., Boyle, M. P. D. & Ormerod, M. G.

(1976) The Destruction of Allogeneic Tumor Cells by Antibody and Adherent Cells from Peritoneal Cavities of Mice. *Cell. Immun.*, **22**, 262.

KATZ, D. & UNANUE, E. R. (1973) Critical Role of Determinant Presentation in the Induction of Specific Responses in Immunocompetent Lymphocytes. *J. exp. Med.*, **137**, 967.

KIESSLING, R., KLEIN, E. & WIGZELL, H. (1975*a*) "Natural" Killer Cells in the Mouse. I. Cytotoxic Cells with Specificity for Mouse Moloney Leukaemia Cells. Specificity and Distribution According to Genotype. *Eur. J. Immun.*, **5**, 112.

KIESSLING, R., KLEIN, E., PROSS, H. & WIGZELL, H. (1975*b*) "Natural" Killer Cells in the Mouse. II. Cytotoxic Cells with Specificity for Mouse Moloney Leukaemia Cells. Characteristics of the Killer Cell. *Eur. J. Immun.*, **5**, 117.

KIESSLING, R., PETRANYI, G., KLEIN, G. & WIGZELL, H. (1975*c*) Genetic Variation of *In vitro* Cytolytic and *In vivo* Rejection Potential of Non-immunised Semi-syngeneic Mice against a Mouse Lymphoma Line. *Int. J. Cancer*, **15**, 933.

KIESSLING, R., PETRANYI, G., KARRE, K., JONDAL, M., TRACEY, D. & WIGZELL, H. (1976) Killer Cells: a Functional Comparison between Natural, Immune T-cell and Antibody Dependent *In vitro* Systems. *J. exp. Med.*, **143**, 772.

LAMON, E. W., WHITTEN, H. D., SKURZAK, H. M., ANDERSSON, B. & LIDIN, B. (1975) IgM Antibody-dependent Cell-mediated Cytotoxicity in the Moloney Sarcoma Virus System: the Involvement of T and B Lymphocytes as Effector Cells. *J. Immun.*, **115**, 1288.

LORD, B. I. (1975) Modification of Granulocytopoietic Cell Proliferation by Granulocyte Extracts. *Boll. Ist. sieroter. milan.*, **54**, 187.

LUNDGREN, G., ZUKOSKI, C. F. & MOLLER, G. (1968) Differential Effects of Human Granulocytes and Lymphocytes on Human Fibroblasts *In vitro*. *Clin. exptl. Immun.*, **3**, 817.

MITCHELL, G. F., HOGARTH-SCOTT, R. S., EDWARDS, R. D., LEWERS, H. M., COUSINS, G. & MOORE, T. (1976) Studies on Immune Responses to Parasitic Antigens in Mice. I. *Ascarissuum* Larvae Numbers and Anti-phosphorylcholine Responses in Infected Mice of Various Strains and in Hypothymic nu/nu Mice. *Int. Arch. Allergy.*, **52**, 64.

NUNN, M. E., DJEU, J. Y., GLASER, M., LAVRIN, D. & HERBERMAN, R. B. (1973) Natural Cytotoxic Reactivity of Rat Lymphocytes against Syngeneic Gross Leukaemia. *Proc. Am. Ass. Cancer Res.*, **14**, 87.

O'TOOLE, C. (1973) Standardization of Microcytotoxicity Assay for Cell-mediated Immunity. *Natn. Cancer Inst. Monogr.*, **37**, 19.

PETRANYI, G. G., BENCZUR, M., ONODY, K., HOLLAN, R. S. & IRANYI, P. (1974) HL-A3, 7 and Spontaneous Lymphocytotoxic Activity. *Lancet*, **i**, 736.

PETRANYI, G. G., KIESSLING, R. & KLEIN, G. (1975) Genetic Control of "Natural" Killer Lymphocytes in the Mouse. *Immunogenetics*, **2**, 53.

PROSS, H.F. & JONDAL, M. (1975) Cytotoxic Lymphocytes from Normal Donors. A Functional Marker of Human Non-T Lymphocytes. *Clin. exp. Immun.*, **21**, 226.

SCHRADER, J. W. & EDELMAN, G. M. (1976) Participation of the H-2 Antigens of Tumor Cells in their Lysis by Syngeneic T-cells. *J. exp. Med.*, **143**, 601.

SENDO, F., AOKI, T., BOYSE, E. A. & BUAFO, C. K. (1975) Natural Occurrence of Lymphocytes showing Cytotoxic Activity by BALB/c Radiation-induced Leukemia RL Male 1 Cells. *J. natn. Cancer Inst.*, **55**, 603.

SHORTMAN, K., WILLIAMS, N. & ADAMS, P. (1972) The Separation of Different Cell Classes from Lymphoid Organs. V. Simple Procedures for the Removal of Cell Debris, Damaged Cells and Erythroid Cells from Lymphoid Cell Suspensions. *J. Immun. Meth.*, **1**, 273.

TAKASUGI, M. & KLEIN, E. A. (1970) A Microassay for Cell-mediated Immunity. *Transplantation*, **9**, 219.

TAKASUGI, M., MICKEY, M. R. & TERASAKI, P. T (1972) Allogeneic Cell-mediated Testing for Human Tumor Antigens. *Natn. Cancer Inst. Monogr.*, **35**, 251.

TAKASUGI, M., MICKEY, R. M. & TERASAKI, P. I. (1973) Reactivity of Lymphocytes from Normal Persons on Cultured Tumor Cells. *Cancer Res.*, **33**, 2898.

TAKASUGI, M., AKIRA, D. & KINOSHITA, K. (1975) Granulocytes as Effectors in Cell-mediated Cytotoxicity of Adherent Target Cells. *Cancer Res.*, **35**, 2169.

WARNER, N. L. (1974) Membrane Immunoglobulins and Antigen Receptors on B and T Lymphocytes. *Adv. Immun.*, **19**, 67.

WOLFE, S. A., TRACEY, D. E. & HENNEY, S. C. (1976) The Induction of "Natural Cytotoxic" Cells by BCG Administration. *Nature*, **262**, 584.

ZARLING, J. M., NOWINSKI, R. C. & BACH, F. H. (1975) Lysis of Leukaemia Cells by Spleen Cells of Normal Mice. *Proc. natn. Acad. Sci. U.S.A.*, **72**, 2780.

ZEMBALA, M., PTAK, W. & HANCZAKOWSKA, M. (1973) The Role of Macrophages in the Cytotoxic Killing of Tumor Cells *In vitro*. I. Primary Immunisation of Lymphocytes *In vitro* for Target Cell Killing and the Mechanism of Lymphocyte-macrophage Co-operation. *Immunology*, **25**, 631.